# Editorial: Non-invasive imaging techniques in children: clinical applications and advances

**DOI:** 10.3389/fped.2025.1675749

**Published:** 2025-08-29

**Authors:** Hualin Yan, Lin Huang, Yan Luo

**Affiliations:** ^1^ Department of Medical Ultrasound, West China Hospital, Sichuan University, Chengdu, China; ^2^ School of Electronic Science and Engineering, University of Electronic Science and Technology of China, Chengdu, China

**Keywords:** non-invasive imaging examination, pediatrics - children, radiology, ultrasonography, technological advancement

**Editorial on the Research Topic**
Non-invasive imaging techniques in children: clinical applications and advances

This topic focuses on the clinical applications and advances of non-invasive imaging techniques in children. However, it is challenging to achieve accurate diagnosis and disease evaluation in children using non-invasive imaging techniques. Many children are unable to remain still or hold their breath during MRI scans, often necessitating sedation or general anesthesia. Besides, children are more sensitive to ionizing radiation from x-rays and CT scans compared to adults. Therefore, adherence to the ALARA (As Low As Reasonably Achievable) principle is essential to minimize unnecessary radiation exposure (1). Compared with MRI or CT, ultrasonography and optical imaging are more friendly to pediatric patients.

The articles included in this special issue explored various aspects of non-invasive imaging in children. Wu et al. evaluated the clinical utility of non-invasive ultrasonography in the diagnosis of disorders of sex development (DSDs) and found that ultrasonography can not only evaluate the internal sex organs, but also provide detailed imaging data of the gonads, including the type, location, size, and morphology, to facilitate clinical management of DSDs. Liu et al. demonstrated that compared with fundus photography, optical coherence tomography (OCT) can evaluate ocular torsion angle effectively in children, offering a novel child-friendly diagnostic tool. Despite involving radiation exposure and potential need for sedation, Liang et al. reported that contrast-enhanced CT remains valuable for the definitive diagnosis of infantile subglottic hemangioma. Lastly, Huang et al. proposed a cohort study to investigate the impact of maternal inflammation on early brain development in infants and young children. They plan to use MRI and electroencephalogram data with image recognition technology to map the early brain development and construct a risk model.

Although the aforementioned four publications represent advances in non-invasive pediatric imaging, they cover only a limited scope of emerging technologies. The cutting-edge non-invasive imaging techniques for pediatric applications are rapidly evolving and notable developments include the followings:

1. Advanced ultrasound techniques: microvascular flow imaging (MFI) enhanced visualization of tumor vascularity, such as in retinoblastoma, providing valuable prognostic insights (2). Ultrasound-derived fat fraction (UDFF) enables quantitative assessment of hepatic fat content based on the combination of attenuation coefficient and backscatter coefficient. A recent multi-center study demonstrated excellent diagnostic performance of UDFF for detecting and grading hepatic steatosis (3);
2. Photoacoustic and optical imaging: photoacoustic imaging is now a mature clinically applicable technology that can quantify tissue oxygen saturation (sO_2_) and hemoglobin levels non-invasively. Multispectral optoacoustic tomography (MSOT) has been applied in various clinical scenarios, such as assessment of Crohn's disease activity (4) or monitoring pediatric inflammatory bowel disease activity (5). Functional near-infrared spectroscopy (fNIRS) has been widely employed in assessing pediatric neurological and psychiatric conditions, such as cerebral palsy, autism spectrum disorder, and attention deficit hyperactivity disorder (6);
3. MRI and CT innovations for children: faster and motion-robust MRI imaging in pediatric patients not only reduces scan times and minimizes the need for sedation or general anesthesia, but also improves the image quality, such as T2-weighted turbo-spin-echo PROPELLER for brain imaging (7) and 4D FreeBreathing MRI technique for abdominal imaging (8). Low-dose CT is needed for pediatrics to reduce the radiation doses. Photon-counting detector CT can reduce the radiation doses and improve imaging quality in pediatric head imaging compared with conventional CT (9);
4. Artificial intelligence (AI) integrated imaging: the pediatric disorder and imaging are distinct from adults, and the AI model developed for adults has not been shown to work consistently in children (10). Recently, several ultrasound-based deep learning models have been developed to improve the accuracy and efficiency of diagnosing pediatric diseases, such as ileocolic intussusception (11) and biliary atresia (12). To ensure the robustness and external validity of AI model, multi-center and internationally diverse patients should be included in the database. For example, a federated learning platform based on international multi-center MRI images achieved accurate segmentation and high classification accuracy in pediatric brain tumors (13).

In summary, accurate diagnosis in pediatric imaging remains challenging due to technical and patient-related factors. However, rapid technological advancements, ranging from improved imaging modalities to AI-integrated analysis, are making pediatric imaging more child-friendly, accurate, and clinically valuable.
